# Low calf circumference is associated with frailty in diabetic adults aged over 80 years

**DOI:** 10.1186/s12877-020-01830-2

**Published:** 2020-10-19

**Authors:** Yun-Xia Zhu, Yue Zhang, Yan-Yan Wang, Chen-Xi Ren, Jun Xu, Xiao-Yan Zhang

**Affiliations:** Department of Geriatrics, Shanghai Jiaotong University Affiliated Sixth People’s Hospital, Road Yi Shan 600, Shanghai, 200233 China

**Keywords:** Calf circumference, Frailty, Aging, Diabetes

## Abstract

**Background:**

Frailty is now seen as a significant factor in older people with diabetes, whose mortality and disability increased. This study aims to investigate the association between calf circumference (CC) with frailty in diabetic adults aged over 80 years.

**Methods:**

A cross-sectional analysis was performed on the data of 426 diabetic adults aged over 80 years. On admission, demographic data and laboratory parameters were recorded. CC was measured on the lower right leg at the point of the maximal circumference. All participants accepted frailty assessments. Frailty was mainly defined using the Fried frailty phenotype criteria.

**Results:**

The CC levels were significantly lower in the frail than the non-frail (26.7 ± 4.0 vs. 31.2 ± 4.0, *P* < 0.001). CC was negatively correlated with the Fried frailty phenotype index (*P* < 0.001). Logistic regression analysis of frailty revealed that age (Odds Ratio (OR), 1.368; 95% Confidential Interval (CI) 1.002–1.869; *P* = 0.049), CC (OR, 0.756; 95%CI 0.598–0.956; *P* = 0.019) were independent impact factors of frailty after adjusting all the potential confounders. Participants with low CC tertile had a significantly higher Fried frailty phenotype index than those with high CC tertiles. The best CC cut-off value for predicting frailty was 29.3 cm, its sensitivity was 75.0%, and the specificity was 78.6%, and areas under the curve (AUC) was 0.786 (*P* < 0.001).

**Conclusions:**

CC was strongly related to frailty in diabetic adults aged over 80 years, suggesting that CC may be helpful for monitoring physical frailty in older adults in clinical and research settings.

**Supplementary information:**

**Supplementary information** accompanies this paper at 10.1186/s12877-020-01830-2.

## Background

Increasing diabetes and aging has become a global social, health, and economic burden, resulting in functional decline and physical disability of old adults. Diabetic patients already have an accelerated aging process that puts them at a higher risk for becoming frailty or frailty-related phenotype at an earlier age [1, 2]. Frailty is a state of increased vulnerability to inner and external stressors and a limited capacity to recover [3]. A study from John Hopkins Medical Institutions involving 5317 participants aged over 65 reported that frailty is present in 25% of patients with diabetes, compared with a prevalence of 6.9% in the whole community-dwelling population [4]. Frailty is emerging as a new complication of diabetes in addition to the traditional microvascular and macrovascular complications leading to considerable disability [5]. Even frail diabetic patients had high mortality than their non-frail counterparts [6, 7].

Due to the focus on the function being more relevant for older patients, a key priority in managing older patients with diabetes is to delay or avoid the appearance of frailty [8]. Frailty is bidirectional, and with appropriate interventions, it can be reversed [9, 10]. It appears to be a dynamic process with several intermediate stages that can improve or worsen over time, highlighting the need to detect it earlier to reduce potential health outcomes. Another question for why detection of frailty in diabetic patents is so essential is these patients whose risk of hypoglycemia increased, suggesting that reducing or even withdrawing hypoglycemic agents may be needed. Thus, early recognition of frailty and early intervention in older adults with diabetes should be critical in their primary care.

A few components of the frailty phenotype, including weight loss, weakness, slowness, reduced physical activity, and exhaustion, are direct symptoms of decreased muscle strength and function. Although the mechanism linking diabetes and frailty are poorly understood, the main factor explaining this link is sarcopenia, an age-related loss of skeletal muscle mass, strength, and function. After 30 years old, human muscle mass decreases at an annual rate of 1–2%. This tendency is accelerated to an annual rate of 1.5–3% after 60 years and even more rapidly over 75 years old [11]. The progressively decreased muscle mass and increased fat mass are various body compositional changes associated with aging [12]. A study showed skeletal muscle mass (OR = 0.159, 95%CI 0.064–0.396) was a protective factor for frailty in 656 elder inpatients aged ≥65 [13]. Calf circumference (CC) can reflect the skeletal muscle mass and is related to humans’ physical function. It has been proven to be a simple marker for sarcopenia and nutritional risk in a few studies [14–16]. CC was negatively related to mortality risk and frailty index in the senior population [17]. A survey from México reported that low CC level is associated with a higher risk of death in frail patients [18].

Type 2 diabetes is known to cause accelerated muscle loss, predominantly in the lower limbs [19]. Insulin resistance, the primary mechanism of type 2 diabetes, has been recognized as a significant risk factor for frailty due to increased chronic inflammatory, impaired endothelial functioning, altered lipid metabolism, and atherosclerosis [20]. A study included 11,527 subjects aged 40–85 years from the National Health and Nutrition Examination Survey (NHANES) showed a significant negative correlation between the CC and homeostatic model assessment of insulin resistance (HOMA-IR) [21]. We hypothesis CC could be a potential indicator of diabetic frailty. As far as we know, there is still a lack of study to investigate CC’s performance in predicting the frailty of diabetes until now.

This study tried to determine the association between CC and frailty in older diabetic adults over 80 years. People more than 80 years are overgrowing in many countries nowadays. These people are not routinely included in clinical studies, predominantly random control trials, due to their comorbidities, polypharmacy, or generally poor prognosis. However, in the real world, they are mainly frail diabetic patients. We primarily concern with the relationship between CC and frailty in this population.

## Methods

### Study population

We screened 1226 consecutive, aged ≥80 years patients admitted to our division between April 2017 through September 2018. The patients who were non-diabetes (*n* = 644), acute diabetes complication (*n* = 12), carcinomatous cachexia (*n* = 37), critical illness (including acute myocardial infarction, acute stroke, severe infection, etc) (*n* = 47), inability to communicate (*n* = 13), bedridden status (*n* = 35), and edema of lower extremity (*n* = 12) were further excluded (Supplemental Figure 1). In this cohort, we had studied that low CC could increase nutritional risk [16], and the malnutrition was an independent risk factor for mortality [22]. In addition, the Nutritional Risk Screening 2002 (NRS2002) score can independently predict the mortality compared with the Mini Nutritional Assessment Short Form (MNA-SF) over more than 2 years of follow-up [23]. In this study, we focused on the CC’s performance in frailty in this cohort.

### Data collection

A questionnaire is used to collect information on participants’ demographic data, chronic disease history, smoking, and alcohol drinking history (Supplemental Table 1). Examining medical records were further used to verify the data.

### Frailty diagnosis criteria

Frailty was mainly defined using the Fried frailty phenotype [4] and detailed according to “Chinese expert consensus on assessment and intervention for elderly patients with frailty” (Supplemental Figure 2). The total frailty index (range: 0–5) was calculated by allocating 1 to positive responses on each of the five components (Weight loss, Slowness, Weakness, Low activity, and Exhaustion). Participants with a score of ≥3 were diagnosed as frailty.

### Anthropometric measurement

Height, body weight, and waist circumference (WC) were measured by standard methods [24]. Body Mass Index (BMI) was calculated as weight divided by height squared (kg/m^2^). The lower right leg’s greatest circumference was measured in patients’ standing position as CC. For each examination, we measured three times to obtain the mean value for further analysis.

### Handgrip strength

The handgrip strength of the dominant hand was the maximum value measured three times with WCS-100 electronic vibrometer. At least 1 min rest was required between each test.

### Laboratory measurements

All the participants took the collection of blood samples after overnight fasting. Serum Albumin, Creatinine, Hemoglobin, glycosylated hemoglobin A1c (HbA1c) levels were tested, as described in our previous publication [16].

### Statistical analyses

The continuous variables following normal distribution were expressed with mean *±* standard deviation. The Student’s t-test was used to measure the difference between groups (BMI, Waist circumference, Calf circumference, Albumin, Hemoglobin, and HbA1c). For non-normal distributed variables, the median / lower–upper quartile range was used. The Mann–Whitney U test was conducted to measure the intergroup difference (Handgrip strength and Creatinine). Categorical variables were showed with frequency percentage, and the chi-square test was conducted for intergroup comparison (Sex). We further applied Pearson (Age, BMI, Waist circumference, Albumin, Hemoglobin, HbA1c, and Fried phenotype frailty Index) or Spearman (Creatinine and Handgrip strength) correlation analysis to analyze the association between CC and other parameters. Multiple logistic regression analysis was performed to determine the risk factors of frailty. We categorized the patients into tertile and further applied the ANOVA test over the Fried frailty phenotype index. The receiver operating characteristic (ROC) curve was used to identify the optimal CC cut-off points to predict frailty. SPSS 21.0 software was used to do all the data analyses.

## Results

### General characteristics of the participants

We enrolled 426 patients (mean age, 86.7 ± 4.3 years; male/female, 305/121) in this study. More than half of the patients were frailty (57.75%, *n* = 246). Table 1 showed the frail and non-frail subjects’ clinical characteristics. The age, Handgrip strength, Albumin, Hemoglobin, HbA1c, and Creatinine were significantly different between the two groups (*P* = 0.048, 0.012, 0.003, 0.014, 0.025, and 0.032, respectively). However, the sex, WC, and BMI were not significantly different between the frail and non-frail (*P* = 0.174, 0.296, and 0.323, respectively).

**Table 1:** Clinical and biochemical characteristics of frail and non-frail old diabetic adults

variable	frail (*n* = 246)	non-frail (*n* = 180)	*P*
Age (years)	88.3 ± 3.4	86.4 ± 3.8	0.048
Sex (male%)	68.89	73.53	0.174
Body mass index (kg/m^2^)	22.89 ± 3.45	24.03 ± 3.39	0.296
Waist circumference (cm)	89.05 ± 11.82	91.96 ± 10.67	0.323
Handgrip strength (kg)	14.43 (6.26–17.53)	20.12 (14.66–23.16)	0.012
Albumin(g/dl)	37.09 ± 6.44	41.00 ± 4.22	0.003
Hemoglobin(g/dL)	107.51 ± 21.49	119.86 ± 17.53	0.014
HbA1c(%)	6.48 ± 0.99	7.12 ± 1.11	0.025
Creatinine (μmol/L)	93.00 (76.50–113.00)	81.00 (64.00–104.00)	0.032

### The difference of CC between frail and non-frail participants

**Fig. 1:**
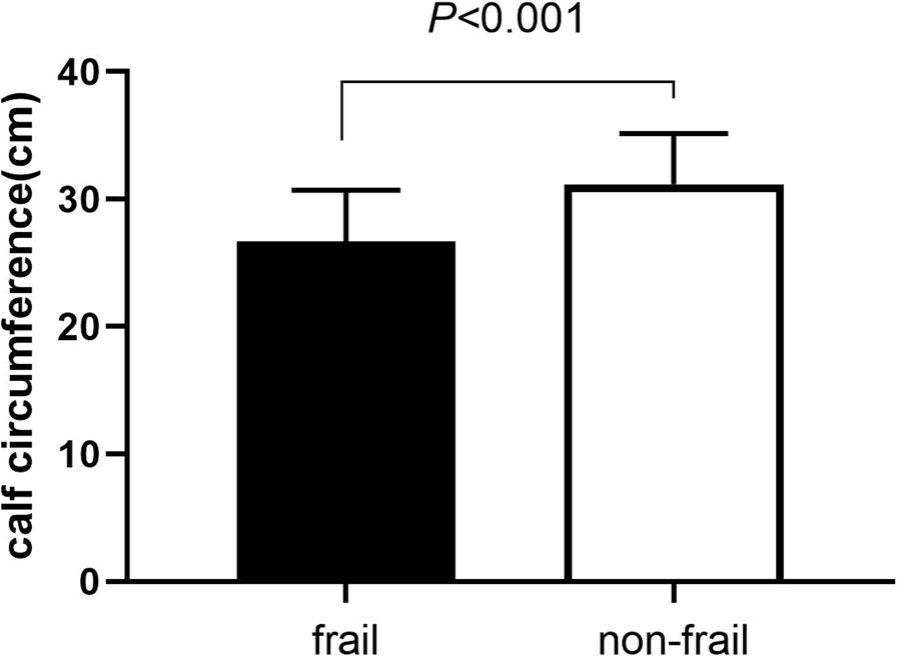
The difference of CC between frail and non-frail participants using Fried phenotype frailty diagnosis criteria

When using Fried frailty phenotype diagnosis criteria, 246 cases were divided as frail. CC levels were significantly different between frail and non-frail group (26.7 ± 4.0 vs. 31.2 ± 4.0, *P* < 0.001) (Fig. 1).

### Factors associated with CC

It was showed in Table 2 that CC was significantly negatively correlated with age and the Fried frailty phenotype index (*P* = 0.041, and < 0.001, respectively). However, CC was significantly positively related to BMI, WC, Hemoglobin, Albumin, HbA1c, and Handgrip strength (*P* = 0.004, < 0.001, < 0.001, < 0.001, < 0.001, and 0.013, respectively).

**Table 2:** Correlation analysis of clinical and biochemical parameters with calf circumference

variable	*r*	*P*
Age	−0.252	0.041
Body mass index	0.469	0.004
Waist circumference	0.540	< 0.001
Handgrip strength	0.688	< 0.001
Albumin	0.531	< 0.001
Hemoglobin	0.484	< 0.001
Creatinine	0.143	0.256
HbA1c	0.371	0.013
Fried phenotype frailty Index	−0.584	< 0.001

### Factors associated with frailty

We calculated the OR (95% CI) of CC associated with frailty in four logistic regression models (Table 3): model 1: adjusted for age and sex; model 2: adjusted for anthropometric indicators (BMI, WC, and HGS); model 3: adjusted for biological markers (Hemoglobin, Albumin, Creatinine, and HbA1c); model 4: adjusted for all the above potential confounders. CC was related to frailty in each adjusted model. In model 4, after adjusting all the confounders, it was revealed that age (OR, 1.368; 95%CI 1.002–1.869; *P* = 0.049), CC (OR, 0.756; 95%CI 0.598–0.956; *P* = 0.019) were independent risk factors of frailty.

**Table 3:** Independent factors for frailty by multivariable logistic regression analysis

variable	Model 1		Model 2		Mode 3		Model 4	
	OR(95%CI)	*P*	OR(95%CI)	*P*	OR(95%CI)	*P*	OR(95%CI)	*P*
Age	1.438 (1.075–1.923)	0.014					1.368 (1.002–1.869)	0.049
Sex	0.659 (0.053–8.347)	0.747					0.780 (0.432–1.409)	0.410
BMI			1.180 (0.903–1.542)	0.225			0.945 (0.789–1.132)	0.538
WC			0.876 (0.713–1.076)	0.207			0.977 (0.934–1.023)	0.320
HGS			0.965 (0.898–1.037)	0.335			0.981 (0.963–1.000)	0.051
CC	0.703 (0.533–0.928)	0.013	0.718 (0.542–0.951)	0.021	0.714 (0.522–0.977)	0.035	0.756 (0.598–0.956)	0.019
Alb					0.748 (0.476–1.176)	0.209	0.890 (0.735–1.079)	0.236
Hb					1.008 (0.932–1.090)	0.839	0.996 (0.952–1.041)	0.834
HbA1c					0.550 (0.278–1.369)	0.120	0.695 (0.233–2.071)	0.514
Cr					1.005 (0.975–1.090)	0.766	1.096 (0.791–1.520)	0.514

*BMI* Body mass index; *WC* Waist circumference; *HGS* Handgrip strength; *CC* Calf circumference; *Alb* Albumin; *Hb* Hemoglobin; *Cr* Creatinine

Model 1 was adjusted for age and sex;

Model 2 was adjusted for BMI, WC, and HGS;

Model 3 was adjusted for Alb, Hb, HbA1c, and Cr;

Model 4 was adjusted for all the above confounders

### The difference of Fried frailty phenotype index according to CC tertile

**Fig. 2:**
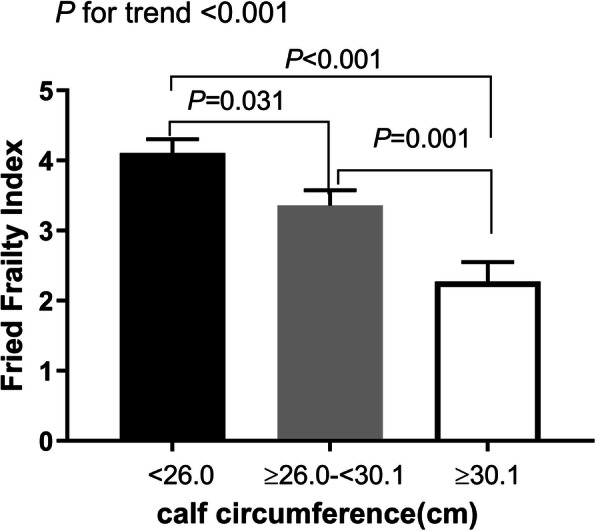
The difference of the Fried frailty phenotype index according to CC tertile

We compared The Fried frailty phenotype index according to CC tertile. Compared with patients in tertile 3 (≥30.1 cm), the Fried frailty phenotype index was significantly higher among participants in tertile 2 (26.0–30.1 cm) and tertile 1 (< 26.0 cm) (P for trend < 0.001) (Fig. 2).

### The cut-off value of CC for frailty

**Fig. 3:**
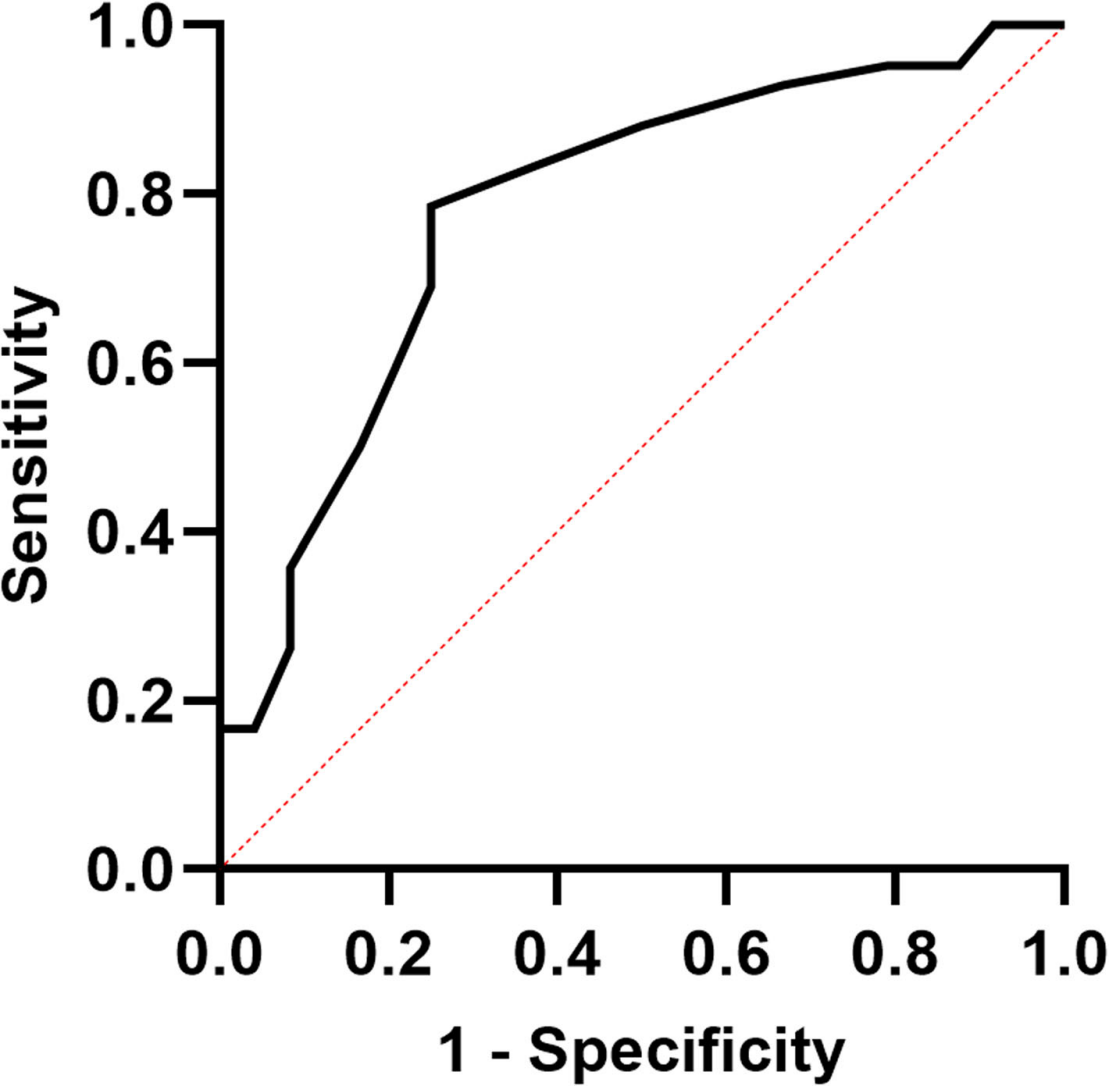
ROC curve analysis of CC for frailty. AUC = 0.786 (*P* < 0.001); 95% CI, 0.669–0.904; CC cut-off point = 29.3 cm; Youden index = 0.536; sensitivity, 75.0%; Specificity, 78.6%

ROC curve analysis was used to find CC’s best cut-off value for identifying frailty in older diabetic patients. The best CC cut-off value was 29.3 cm, the AUC was 0.786 (95% CI: 0.669–0.904), the Youden index was 0.536, with a 75% sensitivity, and 78.6% specificity. (*P* < 0.001) (Fig. 3).

## Discussion

In this study, we provided the evidence for the first time that decreased CC levels were associated with frailty in type 2 diabetic patients aged over 80 years independent of established risk factors. CC levels significantly reduced in type 2 diabetes with frail than their counterparts. CC was negatively correlated with the Fried frailty phenotype index. CC was an independent risk factor of frailty, and the Fried frailty phenotype index increased with a reduction in CC levels. The optimal CC cut-off point for predicting frailty in type 2 diabetic adults aged over 80 years was 29.3 cm in this population.

Frailty is an emerging complication in older people with diabetes [25]. The emergence of frailty may alter the natural history of diabetes from a progressive to a regressive trajectory. Frailty has a triple meaning in older adults with diabetes: a symptom, a prognostic marker, and a therapeutic goal. Our study showed a significant difference in age, Handgrip strength, Albumin, and Hemoglobin between the frail diabetic patients and their counterparts, suggesting that reverse metabolism due to malnutrition in the elderly diabetic patients might be involved. In this study, the old frail type 2 diabetes had a lower HbA1c. There is a consensus that intensive glucose lowering has limited benefits and may be harmful to diabetic older people [26]. It was showed that functional performances over 2 years are better in frail individuals with an HbA1c > 8.0% than those with HbA1c between 7.0–7.9% [27]. A Japanese study showed a relatively lower HbA1c level is a risk factor for frailty, independent of anemia [28]. However, a previous study showed a U-shaped curve of the HbA1c level was a frailty risk factor in patients with type 2 diabetes [29]. Prospective studies need to be done in a massive population to illustrate the relationship between HbA1c and frailty in the old diabetic population.

Anthropometric measurements are usually performed in clinical practice, just like BMI, widely accepted and used. There is scarce commonly used anthropometric measurements on the association with frailty. In this study, there was no difference between frail and non-frail in BMI and WC levels. Usually, at the diabetes early stage, many patients are overweight or obese. However, as people aging, their appetite, caloric need, and energy consumption reduced. They begin to lose weight and become less active, and the potential for frailty occurs. As older adults lose weight and frailty develops, there is an increase in insulin sensitivity, and glucose tolerance improves as visceral fat lost. Indexes of fat distribution such as BMI and WC might be related to adverse health outcomes, mainly due to excessive adiposity in the general population [30]. On the contrary, CC is much more directly associated with a lack of muscle mass than the other anthropometric index. So anthropometric measurements may have different significance in frail old adults.

Muscles play various essential roles in the human body; thus, loss of muscle mass and strength can cause a diverse range of functional disability and metabolic derangements in older adults. The pathogenic linkage between diabetes and frailty potentially includes premature senescence of organ systems, chronic low-grade inflammation, advanced glycosylation end products (AGEs) accumulation, and insulin resistance [31]. Elevation of low-grade systemic inflammation leads to muscle protein breakdown. Diabetes is associated with an increase in inflammatory cytokines, such as tumor necrosis factor and interleukin 6 have detrimental effects on muscle mass, strength, and physical performance in older adults [32, 33]. As a potential diabetic frailty indicator, CC could be easily accessible to predict frailty, especially in communities and primary care settings.

Given the relevance of CC to an individual’s robustness and well-being, identifying clear thresholds for CC and frailty is currently top research and clinical priority. However, anthropometric indices, including CC, tend to vary by age, gender, ethnicity, and environment, making it challenging to determine common values. Asia Working Group for Sarcopenia 2019 (AWGS 2019) recommends that CC could be used to screen sarcopenia, and the cut-off value is < 34 cm for men and < 33 cm for women. While the European Working Group on Sarcopenia in Older People (EWGSOP2) recommends CC may be used as a surrogate for old adults when other muscle mass evaluation methods impossible, the cut-off is < 31 cm [34]. Different values of muscle mass and strength depending on ethnicity support the need for establishing a substantial region-specific cut-off value of CC. In this study, the best CC cut-off value for predicting frailty according to the Fried frailty phenotype was 29.3 cm, with relatively high sensitivity and specificity. In our previous study, we found in aged ≥80 years male, the optimal CC cut-off point for predicting nutritional risk was 29.75 cm; for women was 28.25 cm [16]. Due to the low female percentage (31.11%) in this study, we could not investigate the potential sex-specific effects. It seems the cut-off value of CC to predict diabetic frailty was higher than the cut-off value to predict nutritional risk. That means for diabetic patients, even at relatively higher CC, their frailty risk has increased.

There were some strengths to this study. Firstly, we focused on subjects aged over 80 years, who are routinely excluded in a lot of studies. On the contrary, they are the mainly frail population in the real world. Secondly, some common potential risk factors of frailty had been adjusted, like Albumin, Hemoglobin, and Creatinine. Thirdly, it is a relatively large study of subjects with diabetic patients over 80 years old.

There are many limitations to our study. Firstly, as a cross-sectional and retrospective study, it could not evaluate any cause-effect relationship between CC and frailty. Secondly, there may be sex-specific effects exist between CC and frailty, due to lower female subject ratio in our study, we could not find this. Thirdly, some potential influencing factors on frailty were not considered. For example, all the participants were senior people, some medical history maybe not accurate, like the duration of diabetes. Likewise, the medicine they took were not included in this study. Prospective studies are needed to confirm the association between CC and frailty.

## Conclusions

It is essential to focus on frailty in elderly diabetes patients and intervene in time. As far as we know, this study firstly reported that low CC was strongly associated with frailty in old adults over 80 years, for whom CC may be used as a proxy of frailty. In clinical and research practice, CC may be helpful for monitoring physical frailty in the senior population.

## Supplementary information

**Additional file 1 Supplemental Figure 1**: Flowchart for subjects enrolled in this study. **Supplemental Figure 2**: Frailty diagnosis criteria. BMI: Body Mass Index. MLTA: Minnesota Leisure Time Physical Activity Questionnaire. **Supplemental Table 1**: Participants Assessment Form.

## Data Availability

Not applicable.

## Abbreviations

Alb: Albumin
BMI: Body mass index
CC: Calf circumference
Cr: Creatinine
HbA1c: Glycosylated hemoglobin A1c
Hb: Hemoglobin
ROC: Receiver operating characteristic
WC: Waist circumference
