# Supplementary material for: Low calf circumference is associated with frailty in diabetic adults aged over 80 years
Source: BMC Geriatr. 2020 Oct 19;20:414. doi: 10.1186/s12877-020-01830-2 (PMC7574465; doi:10.1186/s12877-020-01830-2)
Supplement: Supplementary file 1 — Additional file 1 Supplemental Figure 1: Flowchart for subjects enrolled in this study. Supplemental Figure 2: Frailty diagnosis criteria. BMI: Body Mass Index. MLTA: Minnesota Leisure Time Physical Activity Questionnaire. Supplemental Table 1: Participants Assessment Form. [file 12877_2020_1830_MOESM1_ESM.docx]

**Supplemental Figure1: Flowchart for subjects enrolled in this study.**

Total participants aged≥80 years(n=1226)

Final samples (n=426)

Exclusion: (n=156)

acute diabetes complication(n=12)

presence of carcinomatous cachexia(n=37)

critical illness(n=47)

inability to communicate(n=13)

bedridden status(n=35)

edema(n=12)

Exclusion

non-Diabetes (n=644)

Diabetes (n=582)

**Supplemental Figure 2: Frailty diagnosis criteria**

|  | male | female |
| --- | --- | --- |
| Weight loss | Baseline: ≥10 lbs (or 4.5kg) or ≥5% of original weight lost unintentionally in the prior year | |
| Slowness (4.57m) | Height ≤173cm, ≥7s  Height >173cm, ≥6s | Height ≤159cm, ≥7s  Height >159cm, ≥6s |
| Weakness(kg) | Grip strength  BMI≤24kg/m^2^: ≤29  BMI24.1-26.0 kg/m^2^: ≤30  BMI26.1-28.0 kg/m^2^: ≤30  BMI>28 kg/m^2^: ≤32 | Grip strength  BMI≤23kg/m^2^: ≤17  BMI23.1-26.0 kg/m^2^: ≤17.3  BMI26.1-29.0 kg/m^2^: ≤18  BMI>29 kg/m^2^: ≤21 |
| Low activity (MLTA) | <386kcal/week (~walking 2.5h) | |
| Poor endurance; Exhaustion | Center for Epidemiologic Studies Depression Scale (CES-D), NIMH, Score 2-3 points for any question as follows:  How often over the past week they experienced symptoms?   1. I felt that everything I did was an effort. 2. I could not get “going.”   (0 =<1d; 1=1-2d; 2=3-4d; 3=>4d) | |

### **Supplemental Table 1**

### Participants Assessment Form

Name:

Birthday: (mm/dd/year)

ID account:

Assessment date: (mm/dd/year)

Smoking:

current smokers: (active smoking in the last 6 months)

quit:

never:

Drinking:

current drinkers: (active drinking in the last 6 months)

quit:

never:

Education background:

college or above:

high school:

middle school:

elementary school or below:

Diabetes history：Yes/No Duration: year/ Not Sure

Hypertension history: Yes/No Duration: year/ Not Sure

Cerebral infarction history: Yes/No Duration: year/ Not Sure

Chronic obstructive pulmonary disease history: Yes/No

Duration: year/ Not Sure

Coronary heart disease history: Yes/No

Duration: year/ Not Sure

Neoplasms: Yes/No Location：

Duration: year/ Not Sure
